# Multiple cavitary pulmonary metastases from pancreatic cancer diagnosed using transbronchial lung cryobiopsy

**DOI:** 10.1002/rcr2.70031

**Published:** 2024-09-25

**Authors:** Takashi Funasaka, Yasushi Makino, Tomofumi Shibata, Hirotoshi Yasui, Yasutaka Fukui, Mitsuru Odate, Takayasu Ito

**Affiliations:** ^1^ Department of Respiratory Medicine Nagoya University Graduate School of Medicine Nagoya Japan

**Keywords:** cryobiopsy, multiple cavitary lesions, pancreatic cancer, Sjögren's syndrome

## Abstract

Multiple cavitary pulmonary metastases are rare, and cavitary lung lesions have various aetiologies and differential diagnoses. Therefore, radiological diagnosis of lung cavities is extremely difficult. Herein, we report a case of pancreatic cancer with multiple cavitary pulmonary metastases diagnosed using transbronchial lung cryobiopsy (TBLC), that required differentiation from pulmonary lesions of Sjögren's syndrome whose pulmonary manifestation may present as bronchiectasis and cystic change. TBLC may be useful for the diagnosis of multiple lesions because sufficiently large specimens can be obtained to allow pathological evaluation of the lung parenchyma and bronchiolar lesions.

## Graphical abstract

This article reports a case of pancreatic cancer with multiple cavitary pulmonary metastases diagnosed using transbronchial lung cryobiopsy (TBLC), which required differentiation from the pulmonary lesions of Sjögren's syndrome. In this case, computed tomography revealed subpleural reticular shadows and bronchiectasis, in addition to multiple cavities; therefore, we could not precisely diagnose the cavities based on the fibrotic changes in imaging studies. The findings in this case suggest that TBLC is useful for diagnosing multiple miliary lesions.

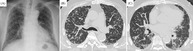

## INTRODUCTION

Hematogenous spread of cancer typically leads to multiple pulmonary metastases scattered throughout both the lungs. In these cases, diagnosis using transbronchial lung biopsy is difficult because of the lack of corresponding bronchi. Transbronchial lung cryobiopsy (TBLC) improves the pathological diagnosis because it provides larger and better‐preserved specimens than conventional forceps biopsies. TBLC is useful for the diagnosis of both endobronchial and localized peripheral pulmonary lesions. However, no study has reported the efficacy of TBLC for the diagnosis of multiple lesions randomly distributed throughout the bilateral lungs. Herein, we report a case of pancreatic cancer with multiple cavitary pulmonary metastases diagnosed using TBLC that required differentiation from pulmonary lesions of Sjögren's syndrome (SS).

## CASE REPORT

**FIGURE 1:**
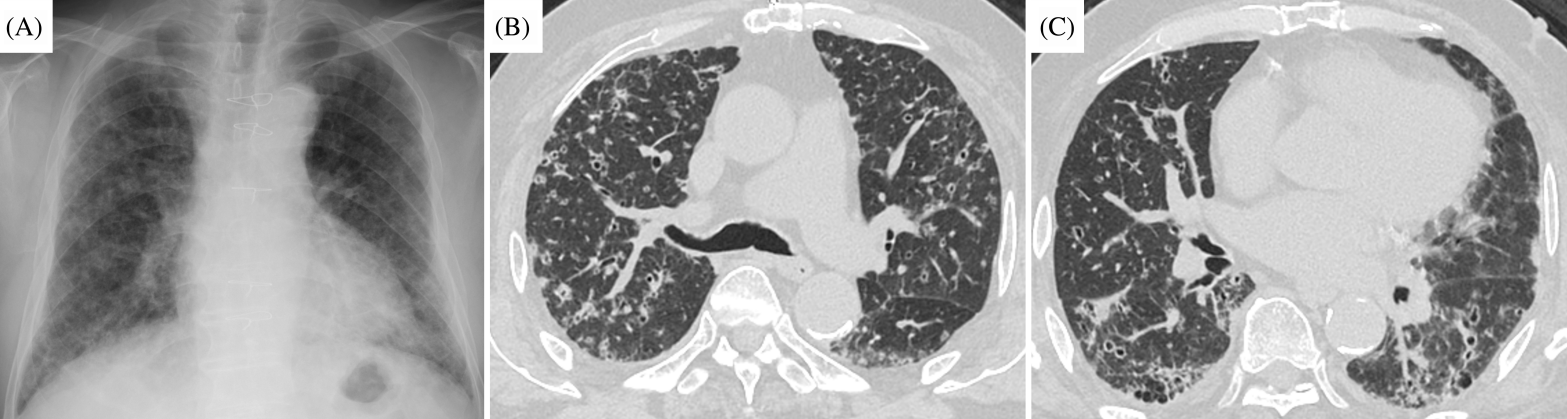
Chest radiograph and Chest computed tomography of the first examination. (A) Chest radiograph showing diffuse bilateral reticular shadows and multiple nodules. (B, C) Chest computed tomography (CT) shows multiple diffuse cavities bilaterally, multiple micronodules (<5 mm), and subpleural reticular shadows in the bilateral lower lobes.

**FIGURE 2:**
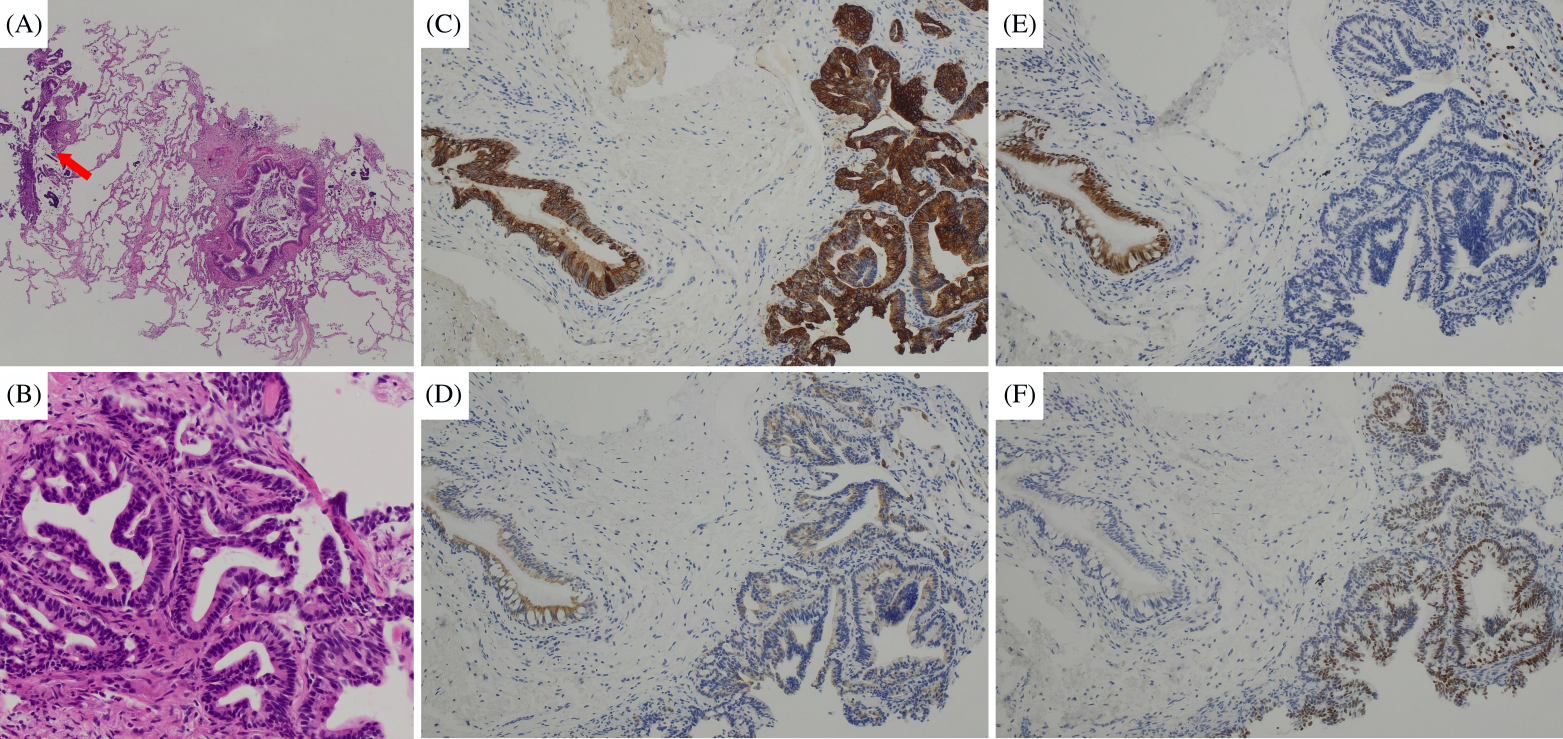
Histopathological findings of transbronchial cryobiopsy. (A) Haematoxylin and eosin (HE) staining shows tumour cells at the edges of the obtained specimen (red arrow) without corresponding bronchi and no abnormal findings in the bronchiole (×25). (B) High‐power magnification shows adenocarcinoma with mucus production along the alveolar surface (×200). (C) CK7 is highly expressed in the atypical glandular cells and bronchiole. (D) CK20 is expressed in the tumour cells and bronchial epithelial cells. (E) TTF‐1 is expressed in the bronchial and alveolar epithelial cells but not in the tumour cells. (F) CDX2 is expressed in the tumour cells but not in the bronchial and alveolar epithelial cells.

An 83‐year‐old man was referred to our hospital with abnormal shadows on chest radiography (Figure 1A). The patient was a former smoker with a smoking history of 45 pack‐years. Medical history included hypertension, type 2 diabetes mellitus, and bladder cancer with no recurrence for >3 years after transurethral resection of bladder tumour and Bacillus Calmette‐Guerin treatment. Chest computed tomography (CT) revealed bilateral multiple diffuse cavities, multiple micronodules, subpleural reticular shadows, and bronchiectasis in the bilateral lower lobes (Figure 1B,C). Peripheral capillary oxygen saturation was 95% on room air. Laboratory tests revealed that the white blood cell count and C‐reactive protein level were not elevated. However, serum levels of Krebs Von den Lungen‐6 (KL‐6) (1375 U/mL) and surfactant protein‐D (SP‐D) (236 ng/mL) were elevated. Additionally, serum autoimmune marker levels were elevated; the antinuclear antibody titre was 1:40 with homogeneous and speckled patterns, and the anti‐SS antibody types A and B levels were 240 U/mL and 236 U/mL, respectively. CT revealed no corresponding bronchi of the multiple pulmonary lesions, and the patient was unable to undergo surgical lung biopsy (SLB) because of poor performance status (PS). Therefore, we performed TBLC using a 1.7 mm cryoprobe with a freezing time of 4 s. Three samples were obtained from the right B8 and B9. Bronchoalveolar lavage was negative for acid‐fast bacilli and Mycobacterium polymerase chain reaction. Histopathological examination of the TBLC specimens revealed adenocarcinoma with no abnormal findings in the bronchioles (Figure 2A,B). Immunostaining of the tumour cells was positive for CK7, CK20, and CDX2 but negative for TTF‐1 tumour (Figure 2B–F). Positron emission tomography‐CT revealed abnormal fluorodeoxyglucose uptake in the pancreatic head and multiple bilateral lung lesions. Endoscopic ultrasound‐guided fine‐needle aspiration of the pancreas revealed pancreatic tumours. Based on these findings, the patient was diagnosed with pancreatic cancer with pulmonary metastasis. He was provided the best supportive care because of his poor performance status (PS).

## DISCUSSION

This case report highlighted two important issues in clinical practice. First, pulmonary metastases may present with multiple cavitary lesions. Second, TBLC is useful for diagnosing multiple miliary lesions.

The lungs are the most common site of cancer metastasis. Common cancers that metastasize to the lung parenchyma include primary lung cancers, colorectal cancers, renal cancers, pancreatic cancers, and breast cancer.^1^ Pulmonary metastasis from pancreatic cancer predominantly presents as rounded solid nodules, and only 7% of pulmonary metastases present as cavitary lesions.^2^ Therefore, we believe multiple cavitary lesions are uncommon imaging patterns of pulmonary metastases from pancreatic cancer. To the best of our knowledge, only one case of pulmonary metastases from pancreatic cancer presenting as multiple cavitary lesions has been reported till date.^3^

The differential diagnoses of lung cavities are wide‐ranging and include malignancies, infections, and connective‐tissue diseases. Therefore, radiological diagnosis of lung cavities is extremely difficult. In this case, CT revealed subpleural reticular shadows and bronchiectasis, in addition to multiple cavities.

We could not precisely diagnose the cavities based on fibrotic changes on imaging, and pathological examination was necessary. In this case, laboratory findings revealed elevated serum levels of KL‐6, SP‐D, and autoimmune markers (antinuclear antibody, and anti‐SS antibody types A and B); thus, the differential diagnosis included connective‐tissue diseases, especially SS whose pulmonary manifestation may present as bronchiectasis and cystic change^4^ and they are the most mimics of lung cavities.^5^

This case report demonstrates that TBLC is useful for diagnosing multiple miliary lesions. In diffuse parenchymal lung disease, TBLC has a higher diagnostic yield compared to conventional forceps biopsy owing to the larger and better‐preserved specimens.^6^ Furthermore, TBLC is useful for the diagnosis of both endobronchial and localized peripheral pulmonary lesions.^7^ However, no study has reported the efficacy of TBLC for diagnosing multiple lesions randomly distributed throughout the bilateral lungs. TBLC specimens are larger and more likely to collect tumour cells randomly distributed in the interstitial areas; therefore, we believe that TBLC is useful for the diagnosis of multiple miliary lesions even if no corresponding bronchi are observed. In this case, the tumour cells were located at the periphery of the specimen, away from the bronchiole, suggesting that transbronchial lung biopsy may not have collected tumour specimens.

Moreover, TBLC may bring more complications, such as pneumothorax and bleeding, but has a lower risk of lethal complications compared with SLB.^8^ In this case, we applied a freezing time of 4 s and used a balloon catheter for bronchial blockade to control bleeding. We believe that TBLC can be safely performed even in elderly patients with poor PS when appropriate methods are utilized.

In conclusion, pulmonary metastases from pancreatic cancer may present as multiple cavitary lesions on imaging, and TBLC is useful for diagnosing multiple miliary lesions. Further studies are needed to elucidate the role of TBLC in diagnosing metastatic tumours with randomly distributed miliary lesions.

## AUTHOR CONTRIBUTIONS

Yasutaka Fukui designed and conceived the manuscript. Yasushi Makino supervised the work. Takashi Funasaka wrote the manuscript with support from Takayasu Ito and Hirotoshi Yasui. Mitsuru Odate and Tomofumi Shibata contributed to the editing of the manuscript. All authors have read and agreed to the published version of the manuscript.

## FUNDING INFORMATION

No funding was received to assist with the preparation of this manuscript.

## CONFLICT OF INTEREST STATEMENT

None declared.

## ETHICS STATEMENT

The authors declare that appropriate written informed consent was obtained for the publication of this manuscript and accompanying images.

## Data Availability

The data that support the findings of this study are available from the corresponding author upon reasonable request.
